# Identifying patients at risk of psychosis: a qualitative study of GP views in South West England

**DOI:** 10.3399/bjgp20X713969

**Published:** 2020-12-01

**Authors:** Daniela Strelchuk, Nicola Wiles, Catherine Derrick, Stanley Zammit, Katrina Turner

**Affiliations:** Centre for Academic Mental Health, Population Health Sciences, Bristol Medical School, University of Bristol; National Institute for Health Research Biomedical Research Centre, University Hospitals Bristol, Weston NHS Foundation Trust, and University of Bristol.; Centre for Academic Mental Health, Population Health Sciences, Bristol Medical School, University of Bristol; National Institute for Health Research Biomedical Research Centre, University Hospitals Bristol, Weston NHS Foundation Trust, and University of Bristol.; Centre for Academic Mental Health, Population Health Sciences, Bristol Medical School, University of Bristol.; Centre for Academic Mental Health, Population Health Sciences, Bristol Medical School, University of Bristol, Bristol; MRC Centre for Neuropsychiatric Genetics and Genomics, Division of Psychological Medicine and Clinical Neuroscience, Cardiff University, Cardiff.; Centre for Academic Mental Health, Population Health Sciences, Bristol Medical School, University of Bristol; National Institute for Health Research Biomedical Research Centre, University Hospitals Bristol, Weston NHS Foundation Trust, and University of Bristol.

**Keywords:** at-risk mental state, general practice, identification, barriers, psychosis, semi-structured interviews

## Abstract

**Background:**

Early intervention in people with an at-risk mental state for psychosis can decrease the rates of transition to psychosis. GPs play a key role in the identification of this patient group but very few studies have explored GPs’ awareness of patients who are at risk of psychosis.

**Aim:**

To explore GPs’ views and experiences of identifying patients with an at-risk mental state for psychosis, and the barriers and facilitators to identification.

**Design and setting:**

In-depth semi-structured interviews were held with GPs working in South West England primary care. The interviews were conducted between March and July 2019.

**Method:**

A topic guide was used to ensure consistency across interviews. This guide was revised to incorporate a definition of the at-risk mental state for psychosis, as after conducting a few interviews it became clear that some GPs were not familiar with this construct. Interviews were audiorecorded and analysed thematically.

**Results:**

A total of 20 GPs were interviewed. Some GPs were not familiar with the concept of being at risk of developing psychosis, and perceived that they may not have the right skills to identify this patient group. Other barriers related to patients not presenting or disclosing psychotic symptoms, and limitations imposed by scarce resources on the structure and provision of NHS services, such as lack of continuity of care and high thresholds for accessing specialised services.

**Conclusion:**

Identifying people at risk of psychosis in primary care is difficult. Provision of GP training, development of policies that support continuity of care, and improved access to specialised services could help improve the identification of this patient group.

## INTRODUCTION

Psychotic illnesses are one of the leading causes of disability worldwide.^1^^,^^2^ The outcome of psychotic illnesses is poor, with most people never making a full recovery.^3^ Yet it is possible to identify those at high risk of developing psychosis using validated criteria and psychometric instruments.^4^ This is important as early intervention can reduce rates of transition to psychosis by approximately 50%.^5^^–^^8^ National Institute for Health and Care Excellence guidelines recommend cognitive behavioural therapy (CBT) for people with an at-risk mental state for psychosis,^9^ but non-specific interventions, such as supportive psychotherapy focusing on social relationships or family problems, may also help.^10^^,^^11^

The prevalence of an at-risk mental state for psychosis is around 1% in the general population.^12^ Individuals with an at-risk mental state for psychosis experience a substantial decline in psychosocial functioning and have either attenuated psychotic symptoms that may last from a few months to 5 years, brief intermittent psychotic symptoms that remit spontaneously, or a strong genetic vulnerability to psychosis.^13^^,^^14^ These people are more likely to be young, male, single, and unemployed.^15^ Approximately 15% and 40% of people with an at-risk mental state for psychosis also suffer from anxiety and depression, respectively.^16^

GPs are usually the first point of contact with health services for people with early signs of psychosis, and play a key role in referring patients to specialised services.^17^ However, identification of people with an at-risk mental state for psychosis is not straightforward given the non-specific nature of its presentation, and the high comorbidity with common mental health problems.^16^^,^^18^

Very few studies have explored GPs’ awareness of the at-risk mental state for psychosis,^19^^,^^20^ and to the authors’ knowledge, no study has explored GPs’ views of these patients in detail, or the difficulties GPs face in identifying this patient group. The aim of this study was to investigate GPs’ views and experiences of identifying patients with an at-risk mental state for psychosis, and the barriers and facilitators to identification.

## METHOD

### Recruitment and sampling

GP practices in South West England were informed about the study via two local Clinical Research Networks (CRNs) between March and July 2019. Practices interested in supporting the study passed on contact details of one or two GPs in their practice, who were willing to be interviewed, to their CRN. These details were then passed to the research team. GPs were informed that the research team were struggling to recruit patients with an at-risk mental state for psychosis to a feasibility trial, and therefore were conducting interviews to better understand GPs’ experiences of identifying and managing these patients.

**Table 1:** How this fits in

Previous research has shown that GPs have limited knowledge about the insidious symptoms of psychosis but little is known about the difficulties that GPs face in identifying patients at risk of psychosis. This study used semi-structured interviews to explore GPs’ experiences of this patient group, and found that some GPs were not familiar with the concept of being at risk of developing psychosis. Whereas this could, in itself, be a barrier to identifying these patients, other barriers were present that related to patients not consulting or disclosing psychotic symptoms, lack of continuity of care, and high thresholds for accessing secondary care services.

Initially the authors aimed to recruit GPs from practices in a catchment area of three early intervention (EI) teams, of which one team was commissioned to work with patients at risk of psychosis. As no referrals were received from practices located in the catchment area of the EI team commissioned to work with these patients, recruitment was extended to the catchment areas of the three other EI teams, two of which were commissioned to work with patients at risk of psychosis. Therefore, GPs working in areas where EI teams were funded to work with these people were recruited and interviewed in the study.

GPs from 21 practices expressed interest in the study. Of these, 16 GP practices were purposefully selected that varied in terms of their deprivation score, list size, and demographic characteristics of patient populations, and in terms of whether or not the practice was in a catchment area containing secondary care services commissioned to work with patients at risk of psychosis. The GPs who had expressed an interest in the study were then interviewed across these 16 practices. The GP practices were reimbursed for the GPs’ time.

### Data collection

A topic guide was used to ensure consistency across the semi-structured interviews. The guide included questions about the recognition, identification, and management of patients with an at-risk mental state for psychosis; advantages and disadvantages of early identification; and facilitators and barriers to early identification. The topic guide was informed by the authors’ experiences of recruiting patients with an at-risk mental state for psychosis to the feasibility study, and findings of other studies on their identification in primary care.^21^

All interviews were conducted by the first author, who is a PhD student with experience of conducting mixed-method studies. Interviews were held between March and July 2019. During the first seven interviews, the first author referred to the patient group of interest as ‘patients at risk of psychosis’ or ‘people who are showing early signs of developing a psychotic illness’. However, as data collection continued it became clear that some GPs were not familiar with this term. Therefore, the topic guide was changed so that at the start of each interview, the first author gave a clear definition of at-risk mental state for psychosis:

*‘People who have mild or short-lived psychotic symptoms such as hearing voices that are just fleeting in nature, or having odd ideas or paranoid beliefs that have not yet formed into strong delusional convictions that are not amenable to rational argument. So these people would not clearly meet the threshold for a psychotic disorder such as schizophrenia, but nevertheless have some symptoms that suggest they might be in the process of developing a psychotic illness.*’

The wording of some of the questions was also changed, that is, patients with an at-risk mental state for psychosis were now referred to as ‘patients with mild or short-lived psychotic symptoms’. In addition, the first author openly asked GPs if they recognised this patient group:

‘Have you come across the concept of an at-risk mental state for psychosis? Is this a patient group you recognise?’

GPs working in areas where EI teams were commissioned to work with patients at risk of psychosis were interviewed with the second, revised, topic guide.

### Analyses

Data collection and analyses were conducted in parallel so that insights from early interviews informed later data collection, and to ensure data collection continued until data saturation was reached, that is, no new themes emerged in the later interviews. All interviews were audiorecorded, transcribed verbatim, and analysed thematically.^22^

The first (having a psychology background) and last (a senior qualitative methodologist) authors independently read and manually coded a sample of transcripts. They then met to discuss their coding and interpretation of data. There was a good level of agreement between the coding of the two authors. Any discrepancies were solved by discussion and resulted in the addition of further codes or clarification of existing ones. After agreeing the new coding frame, all transcripts were uploaded to NVivo (version 12) and coded electronically.

Data under specific codes were then retrieved and summarised in a table where rows presented each interviewee and columns presented the different codes. This enabled the researchers to look across and within the interviews to highlight common themes and deviant cases. Extracts of data and their interpretation were then discussed with the wider team that included the fourth (an academic psychiatrist), second (an epidemiologist), and third (a field worker in primary and secondary care research) authors.

## RESULTS

### Characteristics of GPs interviewed

In total, 20 GPs were interviewed. Of the interviews, 10 were held by telephone and 10 in person at their practice. On average, interviews lasted approximately 30 minutes and eight of the GPs interviewed were females. GPs were aged 32–63 years (mean age: 46.0 years, standard deviation 8.6). The median number of years working as a GP was 14 (interquartile range 12 to 25 years). One of the GPs had an additional qualification in mental health, and another GP had an additional qualification in addictions.

### Recognition of patients with an at-risk mental state for psychosis

When using the first version of the topic guide, some GPs asked for clarification about what was meant by people at risk of psychosis. These GPs were unsure whether the researchers were referring to individuals who had certain risk factors associated with psychosis, such as use of illicit drugs or a trauma history, people who had already had a psychotic illness and were now at risk of relapse, or patients with mild psychotic symptoms:

*‘It’s not something I’ve heard of* … *I didn’t know whether you meant people who might have risk factors* … *or whether you meant people with early symptoms of psychosis.’*

(GP1, female [F], aged 40 years)

After clarifying the meaning, some GPs mentioned that, in their view, these patients were psychotic, rather than at risk of developing psychosis:

*‘To my mind* … *they’re not at risk of psychosis, they have a psychosis, it’s like having a mild broken leg, you either do or you don’t.’*

(GP6, F, aged 44 years)

After revising the topic guide and asking GPs directly whether they recognised this patient group, most GPs reported that they were familiar with the concept of at-risk mental state for psychosis but said they rarely saw these patients. They explained that the patients they had seen with psychotic symptoms either presented in a florid state (that is, very clearly psychotic, with loss of insight), or had recurrent psychosis. GPs also mentioned that it was uncommon for patients to present with isolated mild or short-lived psychotic symptoms, and recognised that in most cases, these mild psychotic symptoms occurred in the context of depression, anxiety, sleep difficulties, use of drugs, life difficulties, or personality disorders. GPs did not refer to these patients as having an at-risk mental state for psychosis but described them as patients with *‘emerging psychosis*’ (GP20, male [M], aged 51 years), or patients with ‘ *soft signs of psychosis’* (GP4, M, aged 32 years).

A number of GPs mentioned that there was no code for the ‘at-risk mental state for psychosis’ but that they did code for specific psychotic symptoms, such as delusions and hallucinations.

Most GPs stated that identifying patients with an at-risk mental state for psychosis was important as it would help patients understand their symptoms better, provide them with information on where to seek help, and improve patients’ outcomes. However, some GPs mentioned potential disadvantages of identifying these patients, such as not being able to offer effective treatment and creating unnecessary worry:

*‘We may be labelling these people* … *but a) not have any effective sort of intervention for them that reduces their risk of progression to psychosis, and b) create, potentially, a lot of unnecessary worry.’*

(GP5, M, aged 35 years)

### Facilitators and barriers to identifying people with an at-risk mental state for psychosis

Clearly, whether a GP recognises this patient group would affect identification of patients. When directly asked for factors that helped or hindered the process of identifying these patients, GPs expanded on their earlier comments and mentioned factors that related to patients’ and GPs’ knowledge, and the NHS.

#### Patient-related facilitators and barriers

Many GPs mentioned that patients with an at-risk mental state for psychosis did not usually consult in primary care, and that people who consulted had usually already transitioned to psychosis.

Most GPs said that they would only see one or two patients at risk of psychosis a year, and a few GPs mentioned that within the last 5 years they had not seen anyone they would classify as being at risk of developing psychosis. However, there were two GPs who reported that they regularly saw such patients. One worked in student health, the other in a deprived area.

GPs felt that some symptoms, such as paranoia, low insight, or low mood could, in themselves, constitute a barrier to consulting as they resulted in patients lacking in motivation to make an appointment.

GPs also thought that patients did not consult because of the stigma associated with psychosis, fear of disclosing psychotic symptoms, and lack of awareness about what constitutes a mental illness and how to seek help:

*‘If it’s mild symptoms and a patient is sort of coping or functioning in the community* … *people may not think of that as being a medical problem* … *there’s probably a lack of awareness as to what symptoms actually are abnormal and therefore merit help, and if it does need help who’s the best person to go for.’*

(GP12, F, aged 54 years)

Some GPs also mentioned that those who did consult did not always feel comfortable disclosing psychotic symptoms. Instead, patients consulted for other symptoms such as depression or anxiety:

‘They won’t come telling you this is what’s going on. You ask people questions to try and establish it and they often lie, not deliberately but because they’re frightened, they don’t want to admit that these things are happening.’

(GP6, F, aged 44 years)

#### GP-related facilitators and barriers

Some GPs mentioned they may not have the skills to identify these patients and may not be asking patients the right questions:

‘I guess there is that barrier of GPs not identifying the people because they don’t have the skills to do that, they don’t have the experience to pick up on that and they’re not asking the right questions.’

(GP2, F, aged 53 years)

It was suggested that one reason for this was because some GPs had limited training in mental health.

Some GPs also mentioned that people with an at-risk mental state for psychosis were not on their radar. Their focus was on more common mental health illnesses, such as depression and anxiety.

A few GPs explained that once a patient met the criteria for a more common mental health illness, they would not always screen for psychotic symptoms. This could be owing to time constraints, or to GPs not remembering or having the knowledge to ask the right questions:

*‘I wonder whether once somebody’s come along with a plausible diagnosis* … *such as depression* … *I wonder whether I don’t ask any questions about psychosis* … *maybe I’m missing them because I’m not asking the right questions, I’m focusing more on the depression and the psychosis side of things isn’t coming out if I haven’t asked the question.’*

(GP12, F, aged 54 years)

Some GPs also reported that as mild psychotic symptoms usually occurred in the context of other mental health illnesses, teasing them apart could be quite difficult:

‘If somebody’s drinking as well then that’s very difficult, how much of it is drugs and alcohol and how much of it is the underlying condition really.’

(GP16, F, aged 49 years)

However, there were some GPs who recognised this patient group. These GPs were more likely to work in areas where secondary care services offered treatment to patients at risk of psychosis, or to work in surgeries with a higher prevalence of young people.

Two GPs also mentioned that making GPs more aware of the effectiveness of treatment, referral routes and availability of services would motivate them to identify these patients:

*‘I wouldn’t be surprised if they* [certain patients] *developed psychosis* … *but* … *a) I’m not aware of any treatment that can prevent progression, b) I don’t think they would be willing to engage with any sort of treatment, and c) I wouldn’t know how to refer anyway.’*

(GP5, M, aged 35 years)

### Structure and provision of services

GPs mentioned that establishing a good rapport would help patients build trust and put them at ease with disclosing psychotic symptoms, as well as help GPs place patients’ symptoms in context, to aid clinical formulation. However, building trust was related to continuity of care and having enough time in consultation, factors that are not under a GPs’ direct influence.

Some GPs also mentioned that booking an appointment was not always easy, and the appointments were too short, particularly as these patients may struggle to bring their psychotic symptoms to the forefront of their narrative.

Many GPs reported the threshold for accessing secondary care as very high, and that patients often fell through the gaps in that they were too severe for primary care, but not severe enough for secondary care. These high thresholds might have deterred GPs from identifying patients with an at-risk mental state for psychosis given the realities of referring:

*‘I can’t think of a patient that I referred to secondary care* … *who has met the threshold* … *It’s not that we’re reluctant to refer people, it’s just that we’re realistic and realise that actually they’re unlikely to get seen if we do try to refer them.’*

(GP5, M, aged 35 years)

A few GPs also mentioned that not being able to offer patients any treatment once they have been identified could be disheartening.

## DISCUSSION

### Summary

GPs may not be familiar with the concept of being at risk of developing psychosis. Some GPs mentioned that they may not be asking the right questions and would benefit from more training on the early symptoms of psychosis. GPs also reported that mild or short-lived psychotic symptoms often occurred in the context of other mental health disorders, which made the identification of these patients difficult. However, there were GPs who recognised this patient group, but reported that potential patients with an at-risk mental state for psychosis rarely consulted in primary care. In addition, GPs also mentioned that patients did not always feel comfortable disclosing psychotic symptoms. Those GPs who worked in areas where secondary care services were commissioned to offer treatment to patients at risk of psychosis were more likely to recognise this patient group.

The challenges of working within a healthcare system where resource limitations impose restrictions on appointment availability and length of consultations, as well as a lack of continuity of care were mentioned as having a negative impact on identifying these patients *.* Yet, GPs felt that being open, non-judgemental, and being able to establish a good therapeutic relationship could facilitate their identification. In psychological therapies, establishing a good therapeutic relationship has been shown to account for approximately 30% of the variation in psychotherapy outcome.^23^

GPs reported that identifying and managing patients with an at-risk mental state for psychosis could improve patients’ outcomes. However, there may be potential disadvantages such as the issue of overlabelling and potentially creating unnecessary worry at a time when GPs had little to offer patients in terms of providing effective interventions or referral to specialist services.

### Strengths and limitations

Both male and female GPs were interviewed, with a range of clinical experience and who worked in areas where secondary care services were or were not commissioned to work with patients at risk for psychosis. Interviewing continued until data saturation had been reached and efforts were made to recruit GPs from the catchment areas of all six EI teams. The authors recognise though that the 21 GP practices that originally expressed an interest in the study, and from which 16 GP practices were purposefully selected, were self-selecting. It may be that GPs with a special interest in mental health were more likely to respond to the study invitation to participate, and this might have biased the present results in terms of including GPs who were perhaps more aware than their peers of this patient group. No difference was noticed in the depth of discussion between the telephone and face-to-face interviews, and research has shown that telephone interviews can gather the same material as those conducted face-to-face.^24^

The original topic guide was revised and GPs given a definition as some GPs were unfamiliar with the concept of at-risk mental state for psychosis. This helped ensure that GPs understood the patient group of interest, but doing so may have sensitised participants to this concept. After changing the topic guide, most GPs said that they were familiar with this patient group. This might be because about half of the GPs interviewed with the second topic guide worked in areas where secondary care services were commissioned to offer treatment to patients at risk of psychosis, but it could also be because providing a definition helped GPs recall patients they had consulted, or encouraged them to give what they thought were more socially desirable answers. Internal bias was minimised in data analysis by double coding some interviews, and discussing results with other clinicians, but the authors recognise that it would have been beneficial to have involved a GP in data analysis.

### Comparison with existing literature

Other studies have shown that GPs may not recognise symptoms of early psychosis.^19^^,^^20^ The presented study extends these findings by highlighting factors that facilitate or hinder the identification of this patient group. The only study that has so far investigated predictive factors of identifying people at risk of psychosis used a semi-structured discussion with GPs to inform the construction of a questionnaire that was later applied to GPs working across England.^21^ That study found that GPs’ subjective norms, that is, a GP’s perception of whether their colleagues identify people with an at-risk mental state for psychosis, and whether other health professionals would approve of them doing so, were the strongest predictor of identifying these patients. The current study used semi-structured interviews and found that the identification of this patient group is a complex process that arises from an interplay of factors related to patients, GPs, and the challenges of working within the NHS.

GPs in the present study reported that they rarely saw patients with mild psychotic symptoms, which is consistent with findings by Simon *et al*.^20^ GPs also mentioned that there was a tendency for patients to consult only after their symptoms had worsened, and potentially transitioned to psychosis. Some support for this comes from a population-based cohort study, which showed that 50% of individuals aged 18 years^25^ and 30% of those aged 24 years^12^ who met criteria for a psychotic disorder had not sought professional help. However, other studies have shown that people with schizophrenia visited their GPs 43% more than controls in the 6 years before their index diagnosis,^26^ and that increasing frequency of consultations in primary care was a strong predictor of psychosis.^27^ This indicates that many people at risk of psychosis are indeed consulting, but the non-specific nature of early symptoms of psychosis, and high comorbidity with anxiety and depression, may hinder their identification as patients with an at-risk mental state for psychosis.^27^^–^^29^

The identification of these patients could be further complicated by the fact that patients who consulted did not always mention their psychotic symptoms. People with an at-risk mental state for psychosis most commonly consult for depression or anxiety.^30^^,^^31^ Therefore, GPs asking patients with depression or anxiety about psychotic phenomena could help identify individuals at risk of psychosis. Short screening tools such as the Primary Care Checklist could guide GPs as to when a specialist assessment might be warranted.^32^

Presence of suicidal behaviour and a pattern of increasing frequency of consultation also appear to be potentially important markers of risk.^27^ Other risk factors associated with an at-risk mental state for psychosis, for example, being a young adult, male, unemployed, with a lower educational level, trauma history, cannabis use, and social isolation,^33^ might also guide GPs on when to screen for psychotic symptoms.

Even though the transition rates to psychosis are quite low (around 20% in the first year),^34^ patients with an at-risk mental state for psychosis have an increased risk of developing other poor outcomes.^35^ If GPs are to feel confident that identifying such patients will improve treatment outcomes, then specific interventions for managing these patients need to be identified.

In the meantime, GPs might be reassured by recent evidence that shows non-specific psychosocial interventions, for example, supportive psychotherapy focusing on social relationships, and assistance with accommodation and monitoring, could also improve patients’ outcomes.^10^^,^^11^ It is possible that some of these interventions may be delivered in primary care, and that shared-care models with input from secondary care services would be beneficial to patients at risk of psychosis. This would be especially relevant for GPs working in areas where secondary care services are not funded to work with these particular patients, and where the duty of care rests with the GP.

In the light of evidence showing that the onset of psychosis can be prevented, it is important that clinicians identify these patients and intervene early. However, the authors are aware that early identification and provision of treatment will be challenging where there are limited resources.

### Implications for research and practice

Clinical guidelines recommend that people who may be at risk of developing psychosis should be referred without delay to specialist services.^9^ However, GPs may not be familiar with this concept, and need more training on how to identify this patient group. Continuity of care is likely to help identify people with an at-risk mental state for psychosis as it improves the therapeutic relationship and may encourage patients to disclose psychotic experiences. Therefore, where possible, GP practices should support continuity of care. At the same time, access to specialist services should be improved, so that once GPs identify potential patients with an at-risk mental state for psychosis, there is a pathway for them to be assessed by specialist services and offered treatment.

Future research should explore how GPs manage patients who are potentially at risk of psychosis, and develop programmes for a better identification and management of this patient group which would take into consideration the limitations of working in resource scarce environments.
